# Removal of a broken acupuncture needle in retroperitoneum by laparoscopy: a case report

**DOI:** 10.1186/s12893-019-0572-y

**Published:** 2019-08-06

**Authors:** Zhen-Hua Liu, Hai-Dong Wang, Xiao Xu, Li-Bo Man

**Affiliations:** Department of Urology, Beijing JiShuiTan Hospital, The 4th Medical College of Peking University, No.96 Hui Nan North Road, Beijing, 100096 China

**Keywords:** Acupuncture, Broken needle, Laparoscopy, Case report

## Abstract

**Background:**

Acupuncture is a famous traditional medicine in China, but the complications caused by broken acupuncture needles have been rarely reported. It seems easy to remove the foreign matters usually, but things become difficulty in special issues. Here, we reported a recently encountered case to provide an important teaching point of treating a chronically retained broken needle in retroperitoneum.

**Case presentation:**

A 42-year-old man presented with a chronically retained broken needle in his body after acupuncture therapy two years ago. However, due to the discomfort at the left back recently and ordinary inconvenience such as security check, he came to our hospital for minimally invasive surgery. He was introduced to our department because the broken needle had migrated from subcutaneous to adipose tissue in retroperitoneum during the two years. Considering the position of the broken needle, the patient was performed by laparoscopy in general anesthesia. The operation time was about 31 min and there were only three 7 mm incisions in the left lateral abdominal wall. The X-ray exam was performed to confirm that the broken needle was removed integrally. The patients begun normal activity at 6 h after surgery and was discharged on the second day after surgery.

**Conclusions:**

Acupuncture is widely used for pain treatment in China, but how to handle the complication of acupuncture needle broken in body are rarely reported. Laparoscopy will be the reasonable choice for treating needles broken in retroperitoneum.

## Background

Acupuncture is a famous traditional medicine in the world and for most people acupuncture is reputed to be safe [1]. As acupuncture and moxibustion are increasingly used in world, their widening acceptance necessitates continual safety assessment. Some complications associated with acupuncture have been described in the literature [2–4]. Witt et al reported that there were 19,726 accidents in 229,230 clinical acupuncture cases, which occupied 8.6% of the total subject pool [5]. The common type of acupuncture involves insertion of very thin needles made of stainless steel, silver, or gold into the subcutaneous soft tissues, and the thin needles are easy to break by inappropriate usage. However, the complications caused by broken acupuncture needles have been fairly minor and how to handle the complication of acupuncture needle broken in body are rarely reported. Wu et al reported only seven events of broken needles in a systematic review from 1980 to 2013, and five cases were under open operations to remove the broken needles [6]. It seems easy to remove the foreign matters, but things become difficulty in special issues. In this case report, we reported a recently encountered case to provide an important teaching point of treating a chronically retained broken needle in retroperitoneum by laparoscopy.

## Case presentation

**Fig. 1:**
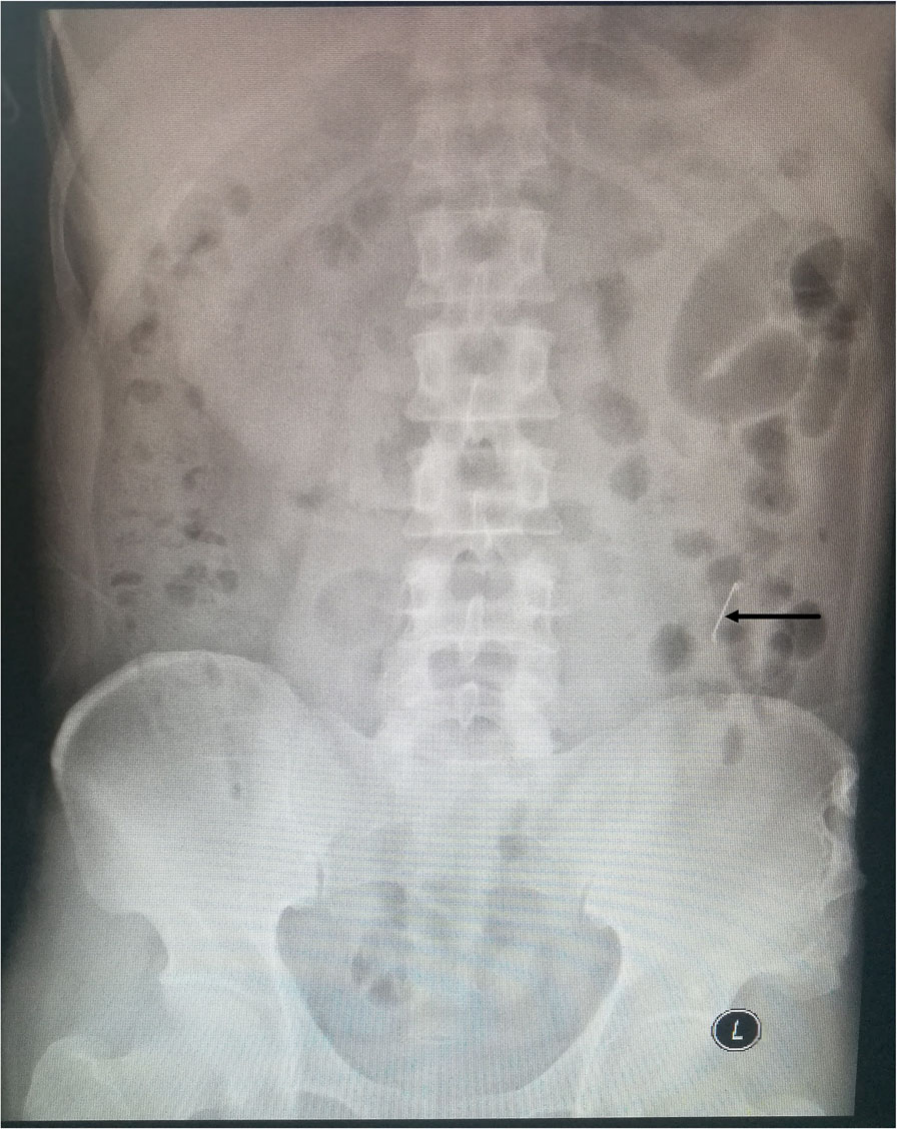
Preoperative Abdominal X-ray. The broken acupuncture needle was showed by arrow

**Fig. 2:**
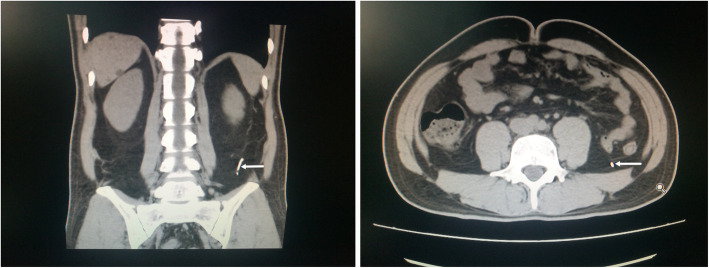
Preoperative Abdominal CT. The broken acupuncture needle was showed by arrow

A 42-year-old man was received acupuncture therapy by an acupuncturist in a Chinese medicine clinic for his back pain two years ago. There were no symptoms for him at the time of the accident happening. However, due to the discomfort at the left back recently and ordinary inconvenience such as security check, he came to our hospital, a trauma center in Beijing, to solve the problem. The patient had no special previously medical history, and there was no positive sign in his back. The patient’s laboratory results were unremarkable, without any findings of leukocytosis. Abdominal X-ray showed that an acicular high density shadow at the level of L4 vertebral in left abdomen (Fig. 1). Abdominal CT scan showed a foreign matter long 2.5 cm in left retroperitoneal fat nearby anterior margin of the quadratus lumbar muscle (Fig. 2). According to the acupuncture therapy history, we basically confirmed that the foreign matter was a broken acupuncture needle retained in the patient body which the acupuncturist had not noticed that after therapy.

**Fig. 3:**
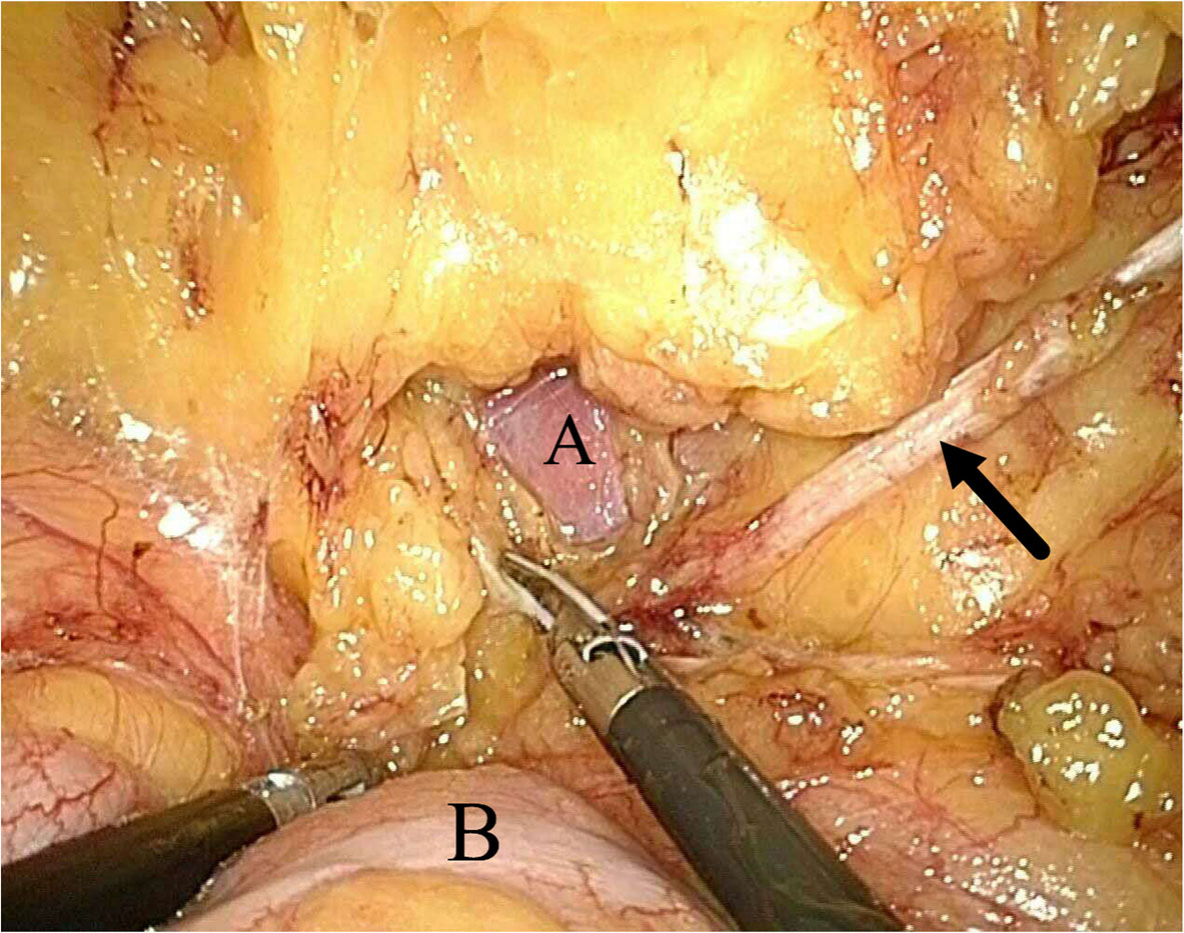
Operation diagram by laparoscopy. **a**, Psoas major muscle; **b**, descending colon; Arrow, the broken needle in a fibrous stripe

**Fig. 4:**
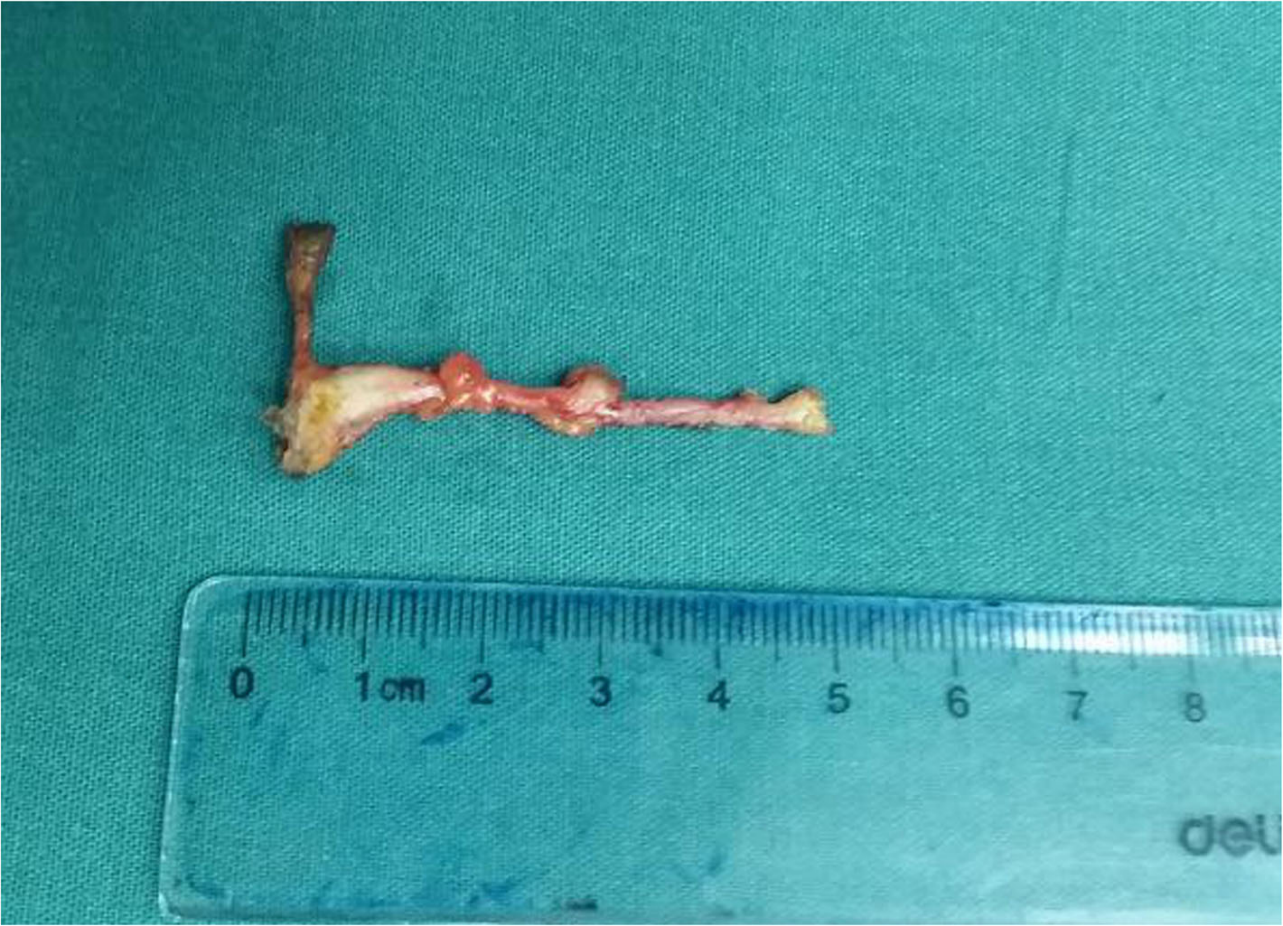
The broken acupuncture needle removed from the patient in fibrous stripe

Considering the position of the broken needle, he was introduced to our department because the broken needle had migrated from subcutaneous to adipose tissue in retroperitoneum during the two years and urologists are more familiar with the anatomical structure of retroperitoneum. The patient was performed by laparoscopy in general anesthesia with lateral position. After establishing pneumoperitoneum, the retroperitoneal fat was isolated until a tough fibrous stripe appeared (Fig. 3). The stripe was completely taken out from the abdomen and confirmed as the broken needle (Fig. 4). An X-ray exam was performed to confirm that the broken needle was removed integrally. The operation time was about 31 min and there were only three 7 mm incisions in the left abdomen region. The patients begun normal activity at 6 h after surgery and was discharged without operative complications on the second day after surgery.

## Discussion and conclusion

Acupuncture has been used in the treatment of pain and other medical ailments for centuries, and its usage is reportedly increasing in the world [7]. Although the frequency of acupuncture associated complications reported appears to be steady over time, the rare adverse effects and complications should also arouse concerns [6]. The most frequent complication of acupuncture treatment is internal organ, tissue, or nerve injury [3, 8]. Wu et al reported seven events of broken needles in a systematic review from 1980 to 2013, and the needles were removed by acupuncturist in two cases and by open surgery in another five cases [7]. Kang reported a case that had a long acupuncture needle broken in the abdomen near the spine in 2015, but the patient was discharged 27 days after the operation due to the perioperative complications [9].

Traditional Chinese needle is designed to be very thin with the tip being acute, allowing it can be migrated deeper [10]. The migrated needles might lead to infection, pain and organ damage. Lazarow et al reported a case that had innumerable chronically retained acupuncture needles, which had migrated throughout her abdomen and pelvis in 2017, and the patient was recommended outpatient follow-up due to the apparent chronicity of the findings and operating difficulty [11]. In our case, the patient was fortunately treated by a minimally invasive surgery with laparoscopy instead of open surgery with large incision. It is noteworthy that the broken needle in our case was also migrated deeper from subcutaneous to adipose tissue in retroperitoneum. Repeated palpation during the residence time could render the broken needle inserted deeper and the needle’s thin and soft property had the inherent disadvantage of being detected among soft tissues. We suggest that the broken needle should be removed at present to avoid serious complications.

Much attention should be paid to the education and training of acupuncturist especially inexperience. There have many training on anatomy and continuation courses on the safety of acupuncture practice for acupuncturists. The acupuncturist does not distract attention during treatment to avoid the ignoring details and shallow needling or not retaining needle is preferred, and the process of treatment should be strictly for unconscious patients unable to cooperate.

In summary, how to remove the broken needles sometimes is a very tough job for surgeon. We performed a laparoscopy surgery for our patient considering the position of the broken needle in retroperitoneum. After establishing pneumoperitoneum, the side peritoneum was cut by ultrasonic knife to separate descending colon, and the retroperitoneal fat was isolated by blunt dissection until the target appeared. The stripe was completely taken out from the abdomen and the side peritoneum was closed by Hemolok. There were two reasons for us to choose the intraperitoneal route, one was that the operating space was enough ample to find the thin needle, the other was the target was always located on the opposite of operation channel to avoid omitting and separate easily. Hereby, laparoscopy will be a reasonable choice to removal needles broken in abdomen.

## Data Availability

All data generated or analyzed during this study are included in this published article. The data can be obtained by corresponding author.

## Abbreviations

CT: Computed tomography
L4: The fourth lumbar
